# Atypical Presentation Versus an Incidental Finding: A Case of Carotid Artery Stenosis

**DOI:** 10.7759/cureus.91724

**Published:** 2025-09-06

**Authors:** Muhammad Shariq Rahemtoola, Hashim Abid, Shariyar A Rahemtoola, Morgan Blake, Khurram Khan

**Affiliations:** 1 General Surgery, North Manchester General Hospital, Manchester University NHS Foundation Trust, Manchester, GBR; 2 Internal Medicine, North Manchester General Hospital, Manchester University NHS Foundation Trust, Manchester, GBR; 3 Internal Medicine, East Lancashire Hospitals NHS Trust, Lancashire, GBR

**Keywords:** arm pain, carotid artery stenosis, central vertigo, paresthesia, posterior circulation syndrome, unilateral headache

## Abstract

Carotid artery stenosis (CAS), a vascular condition that typically presents with symptoms related to the anterior circulation, rarely manifests with posterior circulation symptoms such as vertigo. We present a rare case involving a man in his late 60s who reported vertigo, a chronic unilateral headache, and altered sensation and pain in his left hand, without any signs of anterior circulation involvement. Imaging revealed severe bilateral CAS with no evidence of posterior infarct on MRI. Initial medical management was followed by referral to a vascular clinic and multidisciplinary team (MDT) review. The patient subsequently underwent a successful right carotid endarterectomy and experienced complete resolution of symptoms on postoperative follow-up. This case highlights the importance of recognizing atypical presentations of CAS. Posterior symptoms may, in some cases, be associated with CAS and therefore should not be disregarded during diagnostic evaluation. Multimodal imaging and MDT-guided decision-making were pivotal in the effective management of this complex presentation.

## Introduction

Carotid artery stenosis (CAS) is a significant cause of ischemic stroke, accounting for approximately 10-20% of all cases [1]. CAS typically arises from atherosclerotic plaque at the carotid bifurcation, a site prone to turbulent flow and embolic risk. The progressive arterial luminal narrowing can lead to hypoperfusion or thromboembolism, thereby compromising cerebral blood flow. The most common etiology for CAS is hypertension, diabetes mellitus, hyperlipidemia, atrial fibrillation (AF), smoking, and advanced age [2]. CAS is classified as either symptomatic or asymptomatic. Asymptomatic CAS is most commonly an incidental diagnosis on an imaging scan and is typically managed conservatively with medical therapy. Intervention is considered for severe stenosis (>70%), but evolving evidence suggests that high-risk plaque morphology may also warrant treatment even at moderate stenosis. In contrast, symptomatic CAS tends to present with signs of anterior circulation compromise such as transient ischemic attacks (TIAs), amaurosis fugax, dysphasia or aphasia, hemiparesis, hemi-paraesthesia, and hemiplegia [3]. Posterior circulation symptoms, such as vertigo, limb and gait ataxia, diplopia, and homonymous hemianopia, are classically attributed to vertebro-basilar insufficiency, rather than carotid pathology [4,5]. As a result, CAS is not usually considered in the differential diagnosis when patients present with isolated or predominant posterior symptoms, particularly in the absence of focal neurological deficits. However, anatomical overlap and the presence of collateral circulation can blur the classical distinction between anterior and posterior circulation, meaning that carotid pathology may, in some cases, present with posterior symptoms.

Vertigo is a common presenting complaint in the emergency setting and is most commonly associated with benign vestibular conditions such as benign paroxysmal positional vertigo or vestibular neuritis, especially when isolated and of short duration. However, in the older population, especially with high cardiovascular risk factors, it is important to exclude a cerebral infarct or TIA [6]. In a similar manner, patients presenting with a chronic unilateral headache, intracranial space-occupying lesions, or giant cell arthritis should be excluded. However, when the symptoms are localized and associated with other focal symptoms, vascular pathology should also be considered [7].

We report the case of a 68-year-old male who presented with episodic vertigo on a background of a chronic unilateral headache to illustrate an uncommon clinical presentation of CAS. In addition to contributing to the need to recognize atypical presentations of CAS, this report highlights the importance of maintaining a broad differential diagnosis in patients with posterior circulation symptoms. This is particularly important when risk factors for atherosclerotic disease are present. It also highlights the value of timely imaging, multidisciplinary (MDT) input, and clinical vigilance in preventing delayed or missed diagnoses. This is of importance as delayed or missed diagnosis of symptomatic CAS significantly increases the risk of emboli, thereby causing vascular events such as stroke and TIA. The risk of these events is significantly reduced in timely diagnosis and prompt management of CAS via medical or surgical intervention [3-5].

## Case presentation

A 68-year-old male presented to the emergency department with a one-day history of acute vertigo. The vertigo occurred in discrete episodes lasting 10-20 seconds and was triggered by movement, with approximately 20 episodes before presentation. It had occurred once before and self-resolved. This occurred on a background of a three-month history of intermittent, sharp, and left-sided headache, along with associated symptoms, including left arm pain and tingling in the left hand. The comorbidities were AF, type 2 diabetes mellitus (T2DM), and spinal stenosis. The patient was on bisoprolol 3.75 mg for rate control and apixaban 5 mg twice a day due to a CHA₂DS₂-VASc Score for AF of 3. For T2DM, the patient was on modified-release metformin 1,000 mg twice a day, once daily 10 mg oral dapagliflozin, along with a once weekly semaglutide injection. His HbA1c one month before admission was 66 mmol/mol and had previously been well controlled within the target range. There was no history of myocardial infarction, malignancy, trauma, or fracture. Also, there was no history of TIA or strokes.

**Figure 1:**
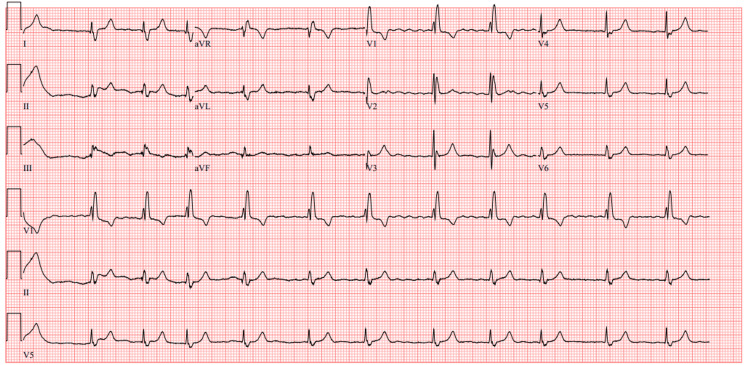
ECG showing atrial fibrillation and right bundle branch block.

**Figure 2:**
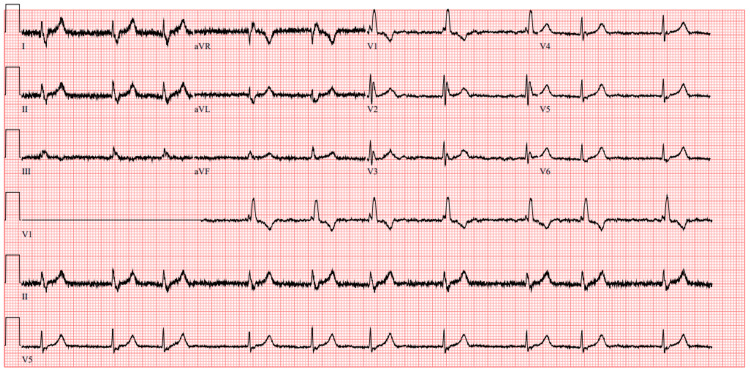
Previous ECG showing atrial fibrillation and right bundle branch block.

Physical examination was unremarkable. Tone, power, and sensation were normal in all four limbs. Cranial nerves were intact. There was no evidence of dysdiadochokinesia, dysmetria, nystagmus, or vertical diplopia. ECG showed atrial fibrillation and right bundle branch block (Figure 1), but without any evidence of notable changes when compared to previous ECGs (Figure 2).

A CT of the head and a CT angiogram of the aortic arch and carotids were performed. There was no acute intracranial pathology or vascular pathology, such as cervical radiculopathy or space-occupying lesions. There was no evidence of an aneurysm or arteriovenous malformation. However, diffuse atherosclerotic changes were noted in the neck vessels, with significant calcified plaque at the level of the carotid bifurcation bilaterally. These findings were corroborated by carotid Doppler ultrasound, which showed mixed, dense, and calcified plaques at the bifurcation and proximal internal carotid arteries, graded as 70-79% on the left and 80-89% on the right.

**Figure 3:**
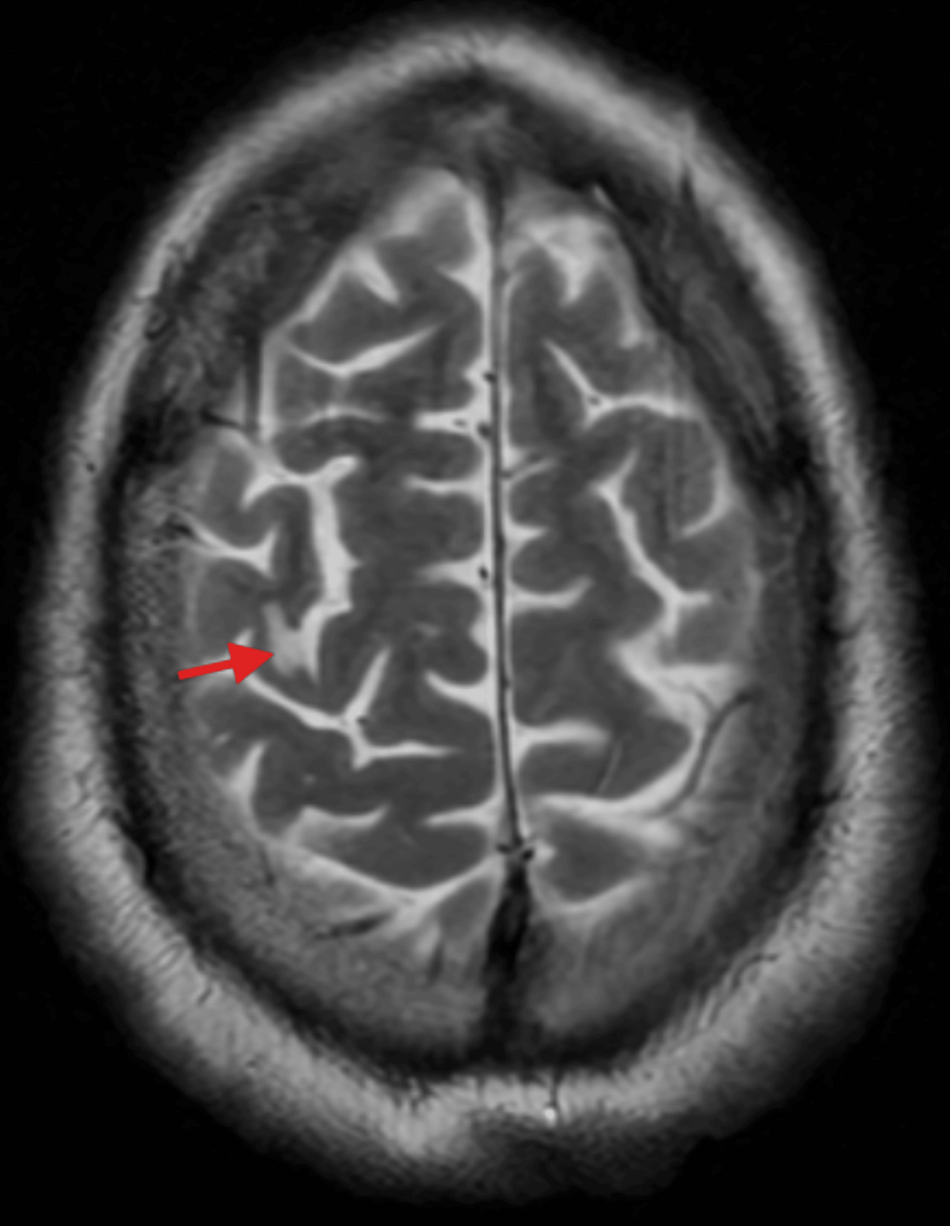
Axial T2-weighted MRI of the brain. The arrow indicates a small, chronic cortical infarct in the right pre-central gyrus (motor strip). No posterior circulation infarct is visible.

MRI of the head was performed to rule out the possibility of a posterior circulation stroke. No acute or established cerebrovascular ischemic changes were seen in the posterior circulatory arteries, though there was a tiny established cortical infarct in the right motor strip (Figure 3).

**Figure 4:**
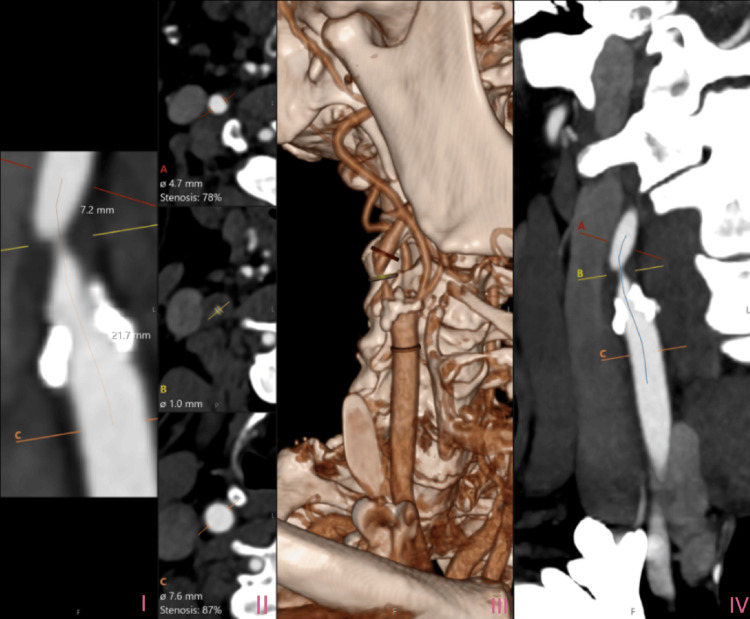
CT angiogram of the right carotid artery. The slide labelled I in the figure shows a longitudinal image of the right carotid artery showing plaque and narrowing. Marked line red shows the distal measurement point. The yellow line shows the point of maximal stenosis, and the orange line shows the proximal reference point. The second slide labelled II shows cross-sectional areas of the right carotid artery at three levels corresponding to the slide I marked A (red), B (yellow), and C (orange). At level marked A (red), the diameter of the lumen is 4.7 mm with 78% stenosis. At level marked B (yellow), the luminal diameter is 1.0 mm, and this is the most stenotic segment. At level C (orange), the luminal diameter is 7.6 mm with 87% stenosis.The third slide labelled III shows a 3D reconstruction of the carotid and vertebral arteries and surrounding anatomy and showing the stenotic areas described above as A (red), B (yellow), and C (orange) in 3D. The last slide labelled IV in the image shows a longitudinal view along the course of the carotid artery. The same stenosis described above as A, B, and C levels marked (matching the axial slices). The blue curved line is a center line of the artery used to follow the vessel path in planning for reconstruction.

**Figure 5:**
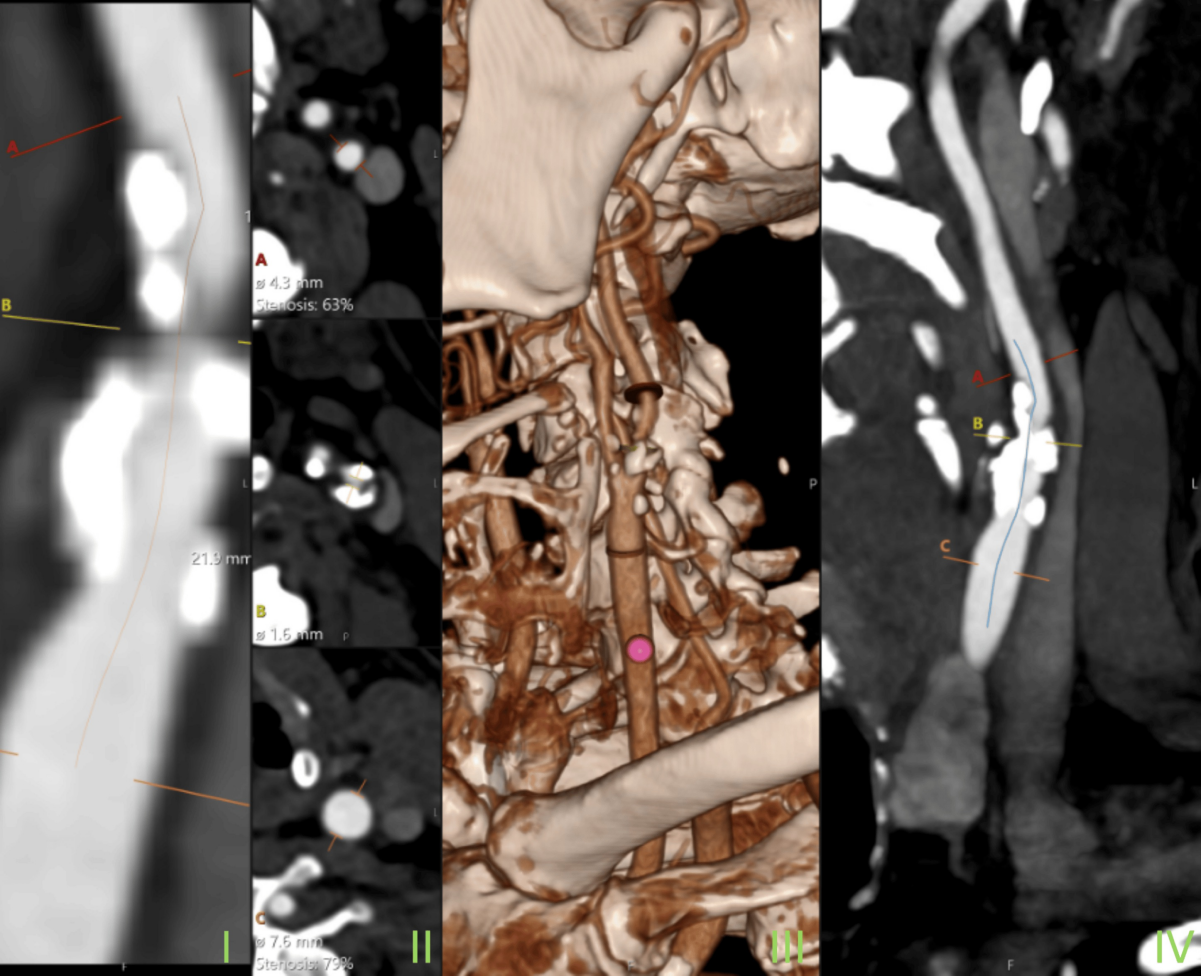
CT angiogram of the left carotid artery. The slide labelled I shows a longitudinal image of the left carotid artery, demonstrating atherosclerotic plaque buildup and luminal narrowing. Marked line A (red) shows the distal measurement point. Line B (yellow) shows the point of maximal stenosis, and line C (orange) shows the proximal reference point. The orange line shows the length of the stenotic segment which is 21.9 mm (orange Line). The slide labelled II shows cross-sectional views of the artery at three levels (A, B, and C), matching locations in Slide I. At level A (red), the luminal diameter is 4.3 mm, corresponding to 63% stenosis. At level B (yellow), the luminal diameter is 1.6 mm, representing the most stenotic region, and at level C (orange), the luminal diameter is 7.6 mm, resulting in 79% stenosis. Slide III shows a 3D reconstruction of the carotid and vertebral arteries and surrounding anatomies and highlights the same stenotic levels, i.e., A (red), B (yellow), and C (orange). Slide IV shows a longitudinal view tracing the entire course of the left carotid artery. Levels A, B, and C are labelled to correlate with images as described in previous slides. The blue curved line represents the center line of the artery for planning reconstruction.

CT angiogram images of the carotid arteries demonstrated the extent of calcified atherosclerotic disease. Multi-planar and 3D reconstructions of the right and left carotids are shown in Figure 4 and Figure 5, respectively.

The patient remained an inpatient until symptoms resolved three days later. During admission, he was commenced on high-dose atorvastatin 80 mg tablet once nightly, and given that he was already anti-coagulated for AF, he was continued on apixaban 5 mg twice a day. Given the three-month history of headache, there was discussion as to whether this represented an atypical presentation of CAS versus an incidental finding. An urgent follow-up with the vascular team was arranged.

At the vascular clinic review, there was no limb weakness or visual or speech abnormalities. Repeat carotid Doppler revealed stenosis of 60-69% in the left carotid bifurcation and internal carotid artery, and 80-89% in the right carotid bifurcation and internal carotid artery. Plaque was characterized as mixed calcified plaque with a disease length of 2.5 cm on the right and 2 cm on the left. Following discussion in the vascular MDT meeting, the patient underwent right carotid endarterectomy. The procedure was uncomplicated, and the patient reported complete symptom resolution at postoperative follow-up.

## Discussion

This case represents a presentation of CAS in which vertigo was the presenting symptom, rather than the more typical findings of CAS such as hemiparesis and dysphasia [5]. A constellation of vertigo, headache, and arm pain would generally suggest a broader differential diagnosis, including a cerebrovascular event or myocardial infarction. In addition, a shorter history with self-resolution, as seen here, would often be associated with a non-vascular vestibular etiology. However, in the context of CAS, vertigo may suggest vertebrobasilar insufficiency secondary to posterior circulation compromise. While it is possible that the carotid disease was an incidental finding, the patient’s significant risk factors, including AF, T2DM, and spinal stenosis, support the interpretation of this case as an atypical presentation of CAS. The likelihood of this interpretation is further strengthened by the extent of bilateral disease demonstrated on imaging.

The comprehensive imaging and diagnostic workup in this case suggests an unusual manifestation of CAS. After ruling out acute cerebrovascular pathology on a CT of the head, a CT angiogram of the aortic arch and carotid arteries, followed by Doppler ultrasound, revealed extensive bilateral plaque burden. Given the possibility of vertebrobasilar insufficiency, an MRI was necessary to exclude posterior circulation infarction. The imaging approach in this case aligns with current guidelines on the investigation of CAS, in which Doppler ultrasound is the most frequently employed modality, followed by CT angiography and magnetic resonance angiography (MRA) [8]. As an inexpensive and non-invasive modality, Doppler is a staple investigation of CAS [9], and one that has been proven to reliably determine the degree of stenosis of more than 70% [10]. CT angiography allows accurate grading of the degree of stenosis and detects features such as calcification and ulceration. However, it cannot differentiate lipid-rich necrotic core from intra-plaque hemorrhage. MRI provides high-resolution images of carotid anatomy, and MRA is considered the gold standard for detecting features such as intraplaque haemorrhage, lipid-rich necrotic core, ulceration, and inflammation. Positron emission tomography/computed tomography (PET/CT) can detect active plaque inflammation but is limited in its ability to characterize plaque morphology [11]. This workup highlights the range of modalities required in both the diagnosis of CAS and the exclusion of other acute differentials. As seen commonly in modern-day practice, the use of such a range of modalities often produces incidental findings. A challenge of modern practice is to balance the need to address incidental findings to prevent subsequent pathology without inundating services with referrals.

This case demonstrates a critical finding, as the patient had extensive CAS and AF and was at a high risk of emboli. Management included high-dose statin and antiplatelet therapy, followed by an MDT discussion and urgent surgical treatment as per the recommendations of the MDT. For patients with asymptomatic CAS, the mainstay of treatment is medical therapy (comprising antiplatelet and statin therapy) and optimization of cardiovascular risk factors (such as those found in this case) [5]. This approach aims to stabilize plaque and reduce the likelihood of a cerebrovascular event.

Surgical intervention, i.e., carotid endarterectomy or carotid artery stenting, depends on symptomatology and stenosis severity. It is standard of care for symptomatic patients with stenosis >70%, as demonstrated in multiple randomized controlled trials (RCTs). However, patients with symptomatic stenosis <50% derive no significant benefit from surgery [2]. The role of surgery in asymptomatic CAS remains debated. While evidence is limited, two large RCTs have shown that carotid endarterectomy for asymptomatic patients with >60% stenosis can yield a modest absolute risk reduction of 5-6% compared to medical therapy [12,13].

The decision to undertake surgical intervention is therefore taken on a risk versus benefit approach, a decision that is aided by patient factors, such as those outlined above, and non-lifestyle elements, such as the degree of stenosis. The latter can be stratified by identifying a variety of plaque characteristics on imaging modalities, such as intraplaque hemorrhage, ulceration, neovascularity, fibrous cap thickness, and the presence of a lipid-rich necrotic core [11]. Subsequent management will include follow-up with the vascular service to monitor for any complications from the procedure, as well as the stability of stenosis.

The changing population demographic, combined with the detailed characteristics derived from these detailed imaging modalities, with the growing availability of interventional radiological procedures, means a paradigm shift is on the horizon. The decision to intervene in any patient with asymptomatic CAS is a risk versus benefit decision, with individual patient risks stratified on the basis of medical imaging. This can result in patients with low-grade stenosis but high-concern plaque characteristics being treated proactively, where previously this would not have occurred [13]. With that said, however, this is an aspect of management that requires further research and evidence, in the form of large prospective or comparative studies and RCTs.

## Conclusions

CAS is a complicated disease with a multifaceted approach to management. At present, the management of CAS must be decided on a risk versus benefit pathway, but to make this decision, a prospective patient must be appropriately risk-stratified. There are several imaging techniques that allow for this, including MRA and PET/CT, which identify plaque characteristics that can be used to predict the risk of ischemic stroke, therefore aiding in key decision-making and improving patient outcomes. In elderly patients presenting with isolated vertigo and cardiovascular risk factors, carotid duplex ultrasound should be considered to avoid missed diagnoses of high-grade stenosis.
